# Charting the Development of Robot-Assisted Social–Emotional Learning: Mapping Its Intellectual Foundations, Thematic Foci, and Evolution

**DOI:** 10.3390/bs16050746

**Published:** 2026-05-11

**Authors:** Wenjia Cui, Kejun Zhang, Zaipeng Zhang, Haoran Cui, Cixian Lv, Taghreed Ali Alsudais, Xinghua Wang

**Affiliations:** 1 Moray House School of Education and Sport, University of Edinburgh, Edinburgh EH8 9YL, UK; s2848738@ed.ac.uk; 2 School of Educational Sciences, Qingdao University, Qingdao 266071, China; zhangkejun1@qdu.edu.cn (K.Z.); zhangzaipeng@qdu.edu.cn (Z.Z.); cuihaoran1@qdu.edu.cn (H.C.); 3 College of Education and Human Development, Princess Nourah bint Abdulrahman University, Riyadh 11564, Saudi Arabia; taalsudais@pnu.edu.sa; 4 Faculty of Applied Sciences, Macao Polytechnic University, Macao, China

**Keywords:** robots, social–emotional learning, scientometric, human–robot interaction

## Abstract

Social and emotional learning (SEL) has become increasingly central to educational policy and lifelong development, while advances in robotics have opened new possibilities for supporting socio-emotional competencies through human–robot interaction. Despite the rapid growth of robot-assisted SEL research, this field remains fragmented, with limited understanding of its intellectual structure, thematic foci, and evolution. To address this gap, this study conducted a scientometric analysis of 241 publications indexed in Web of Science using bibliometric methods. Results indicate a steady growth trajectory, with research concentrated in a small number of core countries driving international collaboration. Influential publications and co-citation patterns reveal a strong foundation in autism-related interventions and child-centered social skill development. Thematic mapping shows that motor themes are dominated by soft skills, autism, and interaction design, while emotion recognition and affective computing form technically mature but specialized streams. Foundational concepts such as human–robot interaction and artificial intelligence remain central yet theoretically evolving. Emerging links between robotics, STEM, and project-based learning suggest ongoing pedagogical expansion. This study maps the intellectual and thematic structure of robot-assisted SEL, showing how robots are emerging as mediational agents in hybrid learning systems while revealing uneven integration and misalignments between technological capabilities and pedagogical foundations.

## 1. Introduction

Social–emotional learning (SEL) refers to the process through which individuals acquire and apply the knowledge, skills, and attitudes necessary to understand and manage emotions, establish positive relationships, make responsible decisions, and navigate social contexts effectively (CASEL, 2003; Murano et al., 2020). Contemporary frameworks, such as those articulated by the Collaborative for Academic, Social, and Emotional Learning (CASEL, 2003), conceptualize SEL as encompassing competencies including self-awareness, self-management, social awareness, relationship skills, and responsible decision-making. A growing body of longitudinal and meta-analytic evidence demonstrates that SEL is associated not only with improved academic achievement but also with long-term psychosocial adjustment, workplace success, and civic engagement across the lifespan (Frye et al., 2024; Murano et al., 2020). In increasingly complex and technologically mediated societies, socio-emotional competencies are also linked to well-being, resilience, and collaborative capacity, positioning SEL as a foundational component of sustainable human development (Cherewick et al., 2021; Domitrovich et al., 2017).

Current approaches to SEL implementation are primarily school-based and typically delivered through structured curricula, teacher-led instruction, and whole-school climate initiatives (Corcoran et al., 2018). Meta-analyses indicate that well-designed, sequenced, active, focused, and explicit (SAFE) programs can yield moderate but significant improvements in both social–emotional skills and academic outcomes (Durlak et al., 2011; Zhong et al., 2025). More recent work emphasizes culturally responsive and context-sensitive models that embed SEL within everyday classroom practices rather than isolated lessons (Lim et al., 2024; Lin et al., 2025). However, traditional approaches face several challenges, including variability in implementation fidelity, heavy reliance on teacher expertise, and challenges in providing individualized, real-time socio-emotional feedback (Chen, 2024; Storey, 2025). Moreover, scaling high-quality SEL interventions across diverse contexts remains difficult, particularly when teacher workload and resource constraints limit sustained delivery (Dang et al., 2025; Sethi & Jain, 2024).

Against this backdrop, researchers have begun exploring robot-assisted SEL as an innovative pathway to support socio-emotional development (e.g., Zhong et al., 2025). Robots, particularly social robots, have been increasingly deployed in educational and therapeutic settings (Belpaeme et al., 2018). They are often physically embodied, programmable agents capable of interacting with humans through speech, gestures, and affective cues (Belpaeme et al., 2018; Yang, 2024). Advances in affective computing and human–robot interaction enable robots to detect, model, and respond to users’ emotional states (D’mello & Kory, 2015; Sethi & Jain, 2024), suggesting potential for personalized socio-emotional scaffolding. Within a sociocultural framework, such robots can be conceptualized as mediational artifacts that participate in learner interaction, potentially supporting the co-construction of socio-emotional understanding through guided engagement. Empirical studies have demonstrated promising applications in autism intervention, emotion recognition training, and classroom engagement (Fang et al., 2025; Scassellati et al., 2012). Nevertheless, the existing body of research remains highly fragmented across disciplines such as robotics, psychology, and education, with limited integration of perspectives and no comprehensive synthesis of its intellectual structure, thematic evolution, and collaboration patterns.

Prior reviews have begun to address this issue. For example, Kewalramani et al. (2024) examined the use of robotics to support SEL in children with autism through a scoping review of 18 studies, while Zhong et al. (2025) investigated a similar topic using a meta-analysis of 20 trials. These studies provide valuable, in-depth insights into how robots can enhance socio-emotional outcomes in children with special needs. However, their methodological focus and population-specific scope limit their ability to capture the broader landscape of robot-assisted SEL research. As a result, they do not provide a comprehensive and generalizable understanding of the field’s overall development, intellectual structure, and thematic evolution across diverse contexts and disciplines. These limitations underscore the need for a bibliometric approach, which is well suited to mapping research trajectories, identifying influential works, and uncovering dominant and emerging themes in an evolving interdisciplinary domain (Aria & Cuccurullo, 2017).

To address these gaps, this study conducts a scientometric analysis and systematic review of robot-assisted SEL research. The dataset comprises 241 publications indexed in the Web of Science (WOS) Core Collection, spanning from 2002 to 2026. First, it traces the evolution of robot-assisted SEL by examining publication growth and geographical distribution, thereby revealing how the field has expanded. Second, it uncovers the intellectual structure of the field through co-citation analysis and examination of highly cited publications and associated research streams that shape current inquiry, and national collaboration network. Third, it analyzes keyword co-occurrence patterns and thematic evolution to capture shifting research priorities, emerging technological orientations, and future directions. By integrating these complementary analyses, this study offers a structured and evidence-based map of robot-assisted SEL research, clarifying what has been established, where the field is converging, and where critical gaps remain. Accordingly, this study addresses the following research questions:

RQ1: What is the overall research trajectory of robot-assisted social–emotional learning research?

RQ2: What are the main intellectual foundations shaping the field of robot-assisted SEL?

RQ3: What emerging themes and thematic evolution can be identified in robot-assisted SEL research?

To address RQ1, we examine the bibliometric profile of the field and its publication growth trends. To answer RQ2, we identify the most influential publications and analyze the corresponding research streams, alongside an examination of collaboration networks across regions. To address RQ3, we conduct word cloud analysis, keyword co-occurrence analysis, and thematic evolution analysis to capture the development of research themes over time.

## 2. Methodology

### 2.1. Tools Selection for Scientometric Analysis

Scientometric analysis enables a systematic and large-scale examination of the intellectual landscape of robot-assisted SEL by processing extensive bibliographic data to identify research trajectories, collaboration patterns, thematic structures, and emerging trends. Given the interdisciplinary nature of robot-assisted SEL, spanning education, psychology, human–robot interaction, and AI, scientometric methods provide a transparent and reproducible approach to synthesizing fragmented knowledge and revealing structural patterns that traditional narrative reviews may overlook (Hallinger & Kovačević, 2019; Mukherjee et al., 2022). Importantly, this study does not aim to replace conceptual or empirical reviews but to complement them by offering a macroscopic, data-driven overview of how the field has evolved over time and across regions.

The *bibliometrix* v5.0 package in R (Aria & Cuccurullo, 2017) was selected as the primary analytical tool due to its flexibility, reproducibility, and comprehensive algorithms for performance analysis, science mapping, clustering, and thematic evolution. To enhance visualization and network interpretation, VOSviewer v1.6.20 was also employed. The combined use of these tools enables a multidimensional and robust mapping of robot-assisted SEL research.

### 2.2. Data Collection

The WOS Core Collection was selected as the data source because of its rigorous indexing standards, detailed citation records, and strong coverage of high-impact journals and conference proceedings (Mongeon & Paul-Hus, 2016). Considering the rapid technological development in robotics and AI, this review focused on publications from 2002 to 2026 to capture contemporary advancements in robot-assisted SEL. Few relevant publications were identified prior to 2002.

A comprehensive search strategy was developed to ensure coverage of both robotics and SEL-related constructs. We adopted the following search query string: Topic = robot* AND Topic = “social and emotional learning” OR “social emotional learning” OR “social emotional” OR “social-emotional” OR “social and emotional” OR “emotional intelligence” OR “social skills development” OR “emotional competence” OR “social competence” OR “emotional regulation” OR “social-emotional development” OR “emotional literacy” OR “social emotional skills” OR “social emotional competence” OR “social emotional abilities” OR “non-cognitive skills” OR “soft skills” OR “character education” OR “resilience training” OR “emotional well-being” OR “relationship skills” AND Topic = “education*” OR “student*” OR “learn*” OR “study*” OR “teach*”. The search was conducted on 6 February 2026.

**Figure 1:**
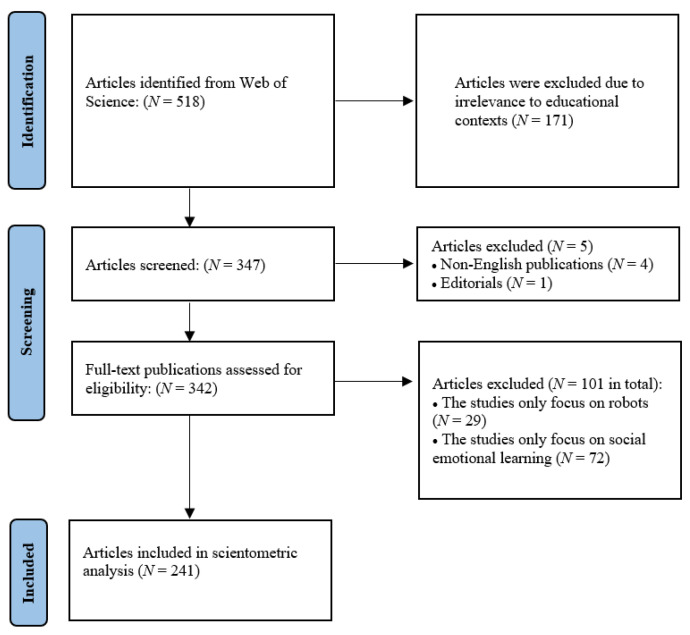
Overview of data collection and filtering process.

Document types were confined to journal articles, proceedings, review papers, etc., excluding editorials. The articles selected were published in English and were peer-reviewed. The initial search yielded 347 records. Two researchers independently screened titles and abstracts based on predefined inclusion and exclusion criteria. The inclusion criterion was that the study must involve the use of robots to support social and/or emotional development. The exclusion criteria were: (a) studies without the explicit focus on social and/or emotional constructs (e.g., emotion recognition, empathy, social interaction, or emotional regulation); (b) studies limited to technical aspects of robotics (e.g., hardware design, navigation, or control systems) without educational or socio-emotional application; and (c) studies addressing SEL without involving any form of robotic or embodied artificial agent. Inter-rater reliability was assessed using Cohen’s kappa (κ = 0.85), indicating strong agreement, and any disagreements were resolved through discussion, with a third reviewer consulted when necessary. Full-text articles were reviewed whenever relevance could not be determined from the titles and abstracts alone. After screening and removing irrelevant records, 241 publications were retained for final analysis (see the Supplementary Materials for the full dataset). The selection procedure is presented in Figure 1.

### 2.3. Data Analysis

*Bibliometrix* was primarily used to conduct performance analysis (e.g., annual publication growth, regional productivity), science mapping (e.g., co-citation networks, collaboration networks), and thematic analysis (e.g., word cloud, keyword co-occurrence, thematic map, and thematic evolution). VOSviewer was used to construct and visualize regional collaboration network and keyword co-occurrence networks to reveal the intellectual and social structure of the field.

For each of the 241 publications, bibliographic information, including titles, authors, affiliations, keywords, abstracts, journal sources, and citation data, was exported from WOS in plain text format. These records were imported into *bibliometrix* for preprocessing, cleaning, and subsequent analysis.

Table 1 summarizes the bibliometric profile of the dataset used in this study. The corpus includes 241 documents published between 2002 and 2026 across 194 sources, indicating that research on robot-assisted social–emotional learning is distributed across diverse journals and conference venues rather than concentrated in a single outlet. The annual growth rate of 7.75% suggests sustained and gradually increasing scholarly attention, while the relatively recent average document age (5.01 years) reflects the field’s emerging and technology-driven nature. With 10,175 cited references and an average of 14.17 citations per document, the literature demonstrates both intellectual grounding and moderate academic impact. Authorship patterns further highlight the collaborative character of the field: 933 authors contributed to the 241 publications, with an average of 4.11 co-authors per document and a low proportion of single-authored works, underscoring its interdisciplinary foundation. International co-authorship accounts for 20.75% of publications, indicating growing global engagement. Finally, the near-equal distribution between journal articles and proceedings papers reveals the strong influence of conference dissemination, which is typical in rapidly evolving domains integrating AI, robotics, and SEL.

## 3. Results

This section presents the results of the scientometric analysis from three parts: (a) the growth trends of robot-assisted SEL publications, (b) the intellectual foundations shaping this field of research, and (c) the thematic focus and evolution.

### 3.1. Growth Trends of Robot-Assisted SEL Publications

**Figure 2:**
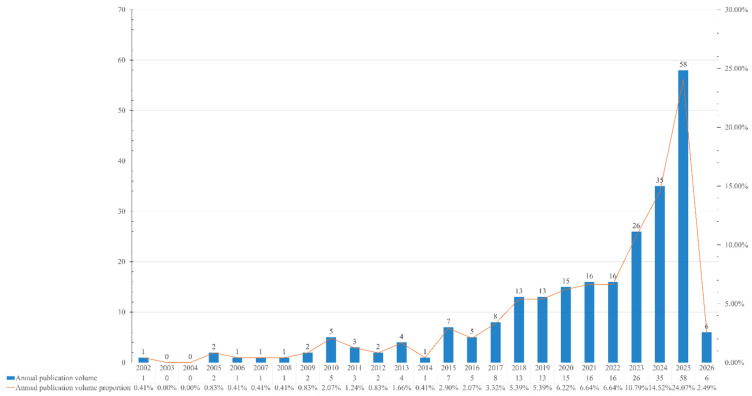
Annual growth of the research publications.

As shown in Figure 2, the publication growth chart reveals a clear developmental trajectory in robot-assisted social–emotional learning research. From 2002 to around 2014, output remained sporadic and low, typically no more than a few publications per year, indicating an exploratory phase in which the field had not yet consolidated. Beginning around 2015, annual production started to increase steadily, marking a transition toward more sustained scholarly engagement. A pronounced acceleration is evident after 2018, with continuous year-on-year growth, culminating in a sharp surge between 2023 and 2025, where publication volume reached its peak. This rapid expansion likely reflects the convergence of advances in AI, social robotics, and growing attention to SEL in educational discourse. Although 2026 shows a lower count, this is most plausibly due to incomplete indexing for the current year rather than an actual decline. Overall, the chart suggests a field that has moved from early experimentation to rapid expansion, entering a phase of intensified and mainstream academic interest.

**Figure 3:**
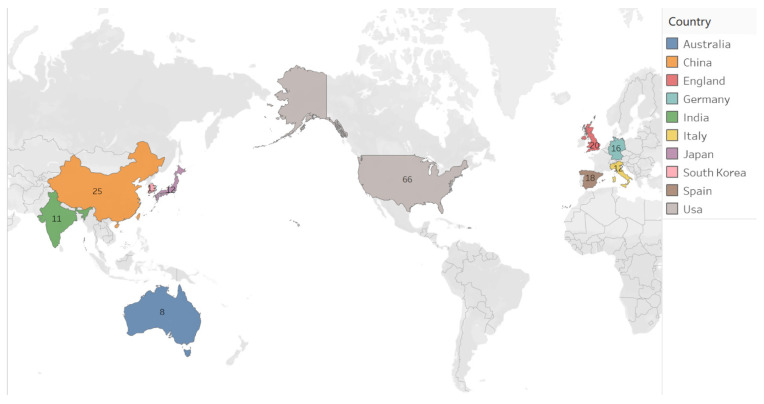
Geographic distribution of the Top 10 countries with the most publications.

The geographical mapping of publications in Figure 3 shows a clear concentration of research output in a limited number of countries. The United States accounts for the largest share of publications, followed by China and England. Additional contributions are visible from South Korea, Spain, Italy, India, and Australia, though at comparatively lower levels. Overall, the distribution indicates that scholarship on robot-assisted SEL is clustered in North America, East Asia, and parts of Europe, with relatively limited representation from other regions such as Africa, South America, and the Middle East.

Taken together, robot-assisted social–emotional learning is a rapidly growing, interdisciplinary field driven by advances in AI and social robotics, with strong collaboration across disciplines and concentration in a few research-leading countries.

### 3.2. Intellectual Foundations Shaping Robot-Assisted SEL Research

The intellectual structure of the SEL research comprises (a) influential publications and associated research streams that shape current inquiry, and (b) collaboration network between different regions.

#### 3.2.1. Influential Publications and Associated Research Streams That Shape Current Inquiry

Table 2 presents the top 10 most cited publications related to robot-assisted SEL. To identify the dominant themes within the top 10 most cited publications, a qualitative thematic synthesis was conducted following principles commonly used in systematic review methodology. First, the full texts of the ten articles were examined to extract information regarding research aims, target populations, intervention characteristics, and reported socio-emotional outcomes. Particular attention was paid to how each study conceptualized and operationalized social–emotional learning (e.g., emotional recognition, empathy, social interaction skills, relational engagement). Coding was conducted independently by two researchers using an inductive approach. Inter-rater consistency (κ = 0.82) was ensured through regular comparison of coding results, with discrepancies discussed and resolved through consensus. In the second step, an inductive coding process was applied to cluster studies with shared conceptual emphases. The coding scheme was iteratively refined through multiple rounds of comparison and consolidation to ensure that themes were internally coherent and externally distinct (see Table S1 for the final coding framework). Finally, each paper was assigned to a single dominant theme based on its primary contribution to robot-assisted SEL, ensuring mutual exclusivity and conceptual clarity across themes. To enhance the robustness of the analysis, the final themes were reviewed and validated through team discussion.

**Theme 1: Robot-assisted development of emotional recognition and social understanding.** This theme includes studies that directly target emotional recognition and socio-cognitive development as core SEL competencies. Cabibihan et al. (2013) provided a foundational review demonstrating how social robots can scaffold emotional expression and social communication in children with autism, establishing the theoretical rationale for robot-mediated socio-emotional intervention. Pop et al. (2013) empirically showed that interaction with the humanoid robot Probo improved children’s identification of situation-based emotions, directly addressing emotional awareness. Marino et al. (2020) reported improvements in social–emotional understanding following structured intervention, while So et al. (2020) observed gains in joint attention and functional play behaviors, which are closely tied to early social cognition and perspective-taking. Together, these studies position robots as structured mediators for strengthening emotional recognition and social understanding, which are foundational elements of SEL.

**Theme 2: Relational and affective mechanisms enabling socio-emotional engagement.** This theme focuses on how robots’ emotional expressiveness, embodiment, and perceived empathy facilitate socio-emotional engagement. Fan et al. (2017) examined human perceptions of emotional intelligence in artificial agents, demonstrating that perceived affective competence shapes trust and acceptance. Kim and Tscholl (2021) highlighted the importance of embodied interaction in shaping children’s emotional meaning-making during engagement with social robots. Leite et al. (2015) showed that emotionally expressive storytelling robots influence participation patterns differently in individual versus group contexts, underscoring how affective design mediates social interaction. These studies collectively suggest that robot-assisted SEL is enabled not only by instructional design but by relational cues and emotional signaling that foster trust, engagement, and social presence.

**Theme 3: Structured social skill training and guided educational integration.** The third theme emphasizes structured practice and guided development of social skills within therapeutic and educational contexts. Giannopulu and Pradel (2012) introduced a child–robot–therapist interaction model, demonstrating how mediated scaffolding enhances reciprocal communication and structured participation. Mengoni et al. (2017) proposed a randomized controlled trial to rigorously evaluate robot-assisted improvement of social skills in children with autism, reflecting methodological advancement in assessing SEL-related behavioral outcomes. Extending beyond clinical contexts, Kewalramani et al. (2021) examined the use of robotic toys in early childhood education, showing how robots can support communication, collaboration, and emotional expression in everyday learning environments. Together, these studies conceptualize robot-assisted SEL as a structured environment for practicing and consolidating social interaction skills through guided facilitation.

#### 3.2.2. Regional Collaboration Network

**Figure 4:**
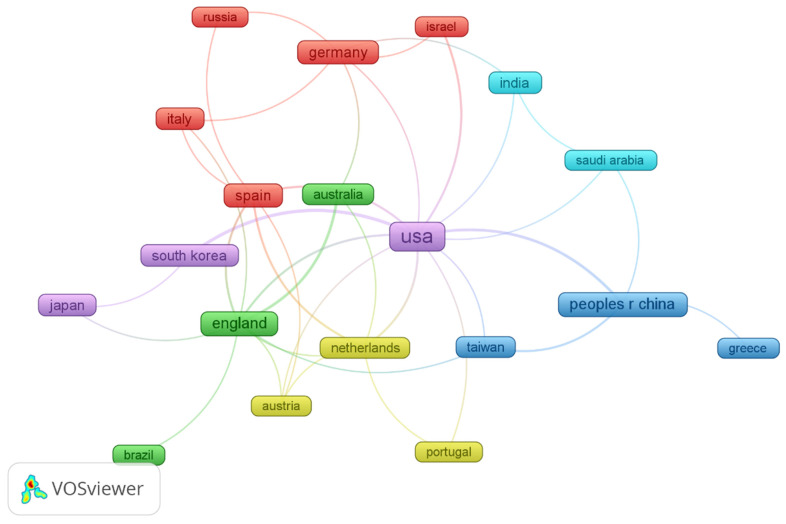
Regional collaboration network.

Figure 4 illustrates the regional collaboration network in robot-assisted SEL research using association strength normalization (minimum documents ≥ 3; 23 regions; minimum cluster size = 1). The network displays a clear hub-and-spoke structure, with the United States positioned at the center of the global collaboration system. The USA maintains strong and multiple links with European countries such as England, Germany, Italy, Spain, and the Netherlands, as well as with Asia-Pacific partners including mainland China, South Korea, and Australia. This pattern suggests that the United States functions as a major bridging node, facilitating cross-regional knowledge exchange and integrating otherwise separate national research communities.

At the same time, several regional sub-clusters are evident, as indicated by the same color in Figure 4. A European cluster (e.g., Germany, Italy, Spain, Russia) demonstrates relatively dense intra-regional collaboration, while England connects both within Europe and outward to regions such as Australia and Brazil. In Asia, mainland China forms a collaboration group with Greece, and India is closely linked with Saudi Arabia, indicating emerging regional partnerships.

Overall, robot-assisted SEL is evolving from a technology-centered approach to a relational, context-sensitive practice in which robots act as socio-emotional mediators embedded within teacher-guided learning environments, while the field itself remains globally collaborative but structurally uneven, relying on a small number of leading regions to drive innovation and knowledge exchange.

### 3.3. Thematic Focus and Evolution in the Field of Robot-Assisted SEL Research

Thematic focus primarily refers to the areas that scholars have been working on for years. Thematic evolution, on the other hand, is related to the changes in research topics (Agbo et al., 2021). Utilizing the *bibliometrix* package in R and VOSviewer, this study conducted multiple rounds of analyses, including word cloud, keyword co-occurrence, and thematic evolution. The keywords were set to author-defined keywords which reflect the specific concepts, ideas, and research focuses as intended by the authors themselves and provide a standardized and consistent way of categorizing and indexing research articles.

#### 3.3.1. Word Cloud Analysis

**Figure 5:**
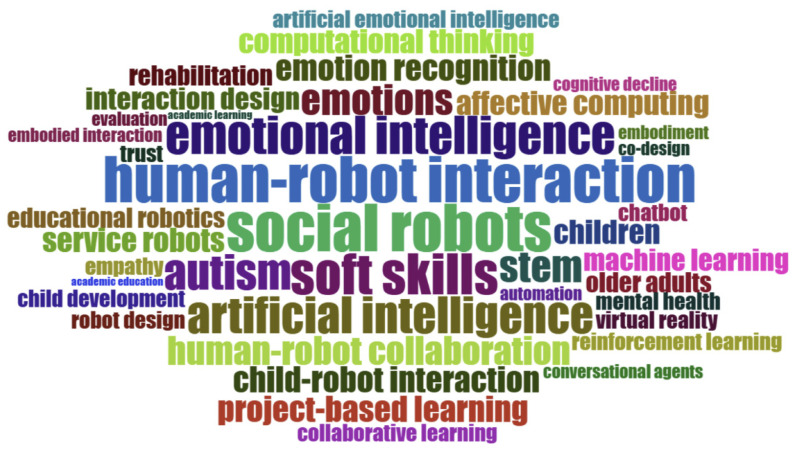
Word cloud in the field of robot-assisted SEL.

The word cloud in Figure 5, which was generated using *bibliometrix*, reveals several clear and dominant patterns in robot-assisted SEL research. Most prominently, large terms such as *human–robot interaction*, *social robots*, *emotional intelligence*, *artificial intelligence*, and *emotions* indicate that the field is primarily centered on emotionally intelligent robotic systems and their interactive functions. The strong visibility of *emotion recognition*, *affective computing*, and *machine learning* further highlights the technological backbone that enables robots to detect and respond to learners’ affective states. At the same time, frequent terms like *children*, *autism*, *child–robot interaction*, and *soft skills* suggest that research is largely child-focused and often situated in developmental or special education contexts. Finally, the presence of *educational robotics*, *collaborative learning*, *project-based learning*, and *STEM* reflects the integration of robots into structured learning environments aimed at fostering socio-emotional competencies. Overall, the most obvious pattern is a convergence of affective AI technologies with child-centered educational and therapeutic applications.

#### 3.3.2. Keywords Co-Occurrence Analysis

Keyword co-occurrence analysis provides a more detailed understanding of the knowledge base composition of robot-assisted SEL compared to co-citation analysis, as it relies on keywords identified within the documents (Hallinger & Kovačević, 2019). This method generates a network of interconnected themes that depict the conceptual landscape of current research on robot-assisted SEL.

**Figure 6:**
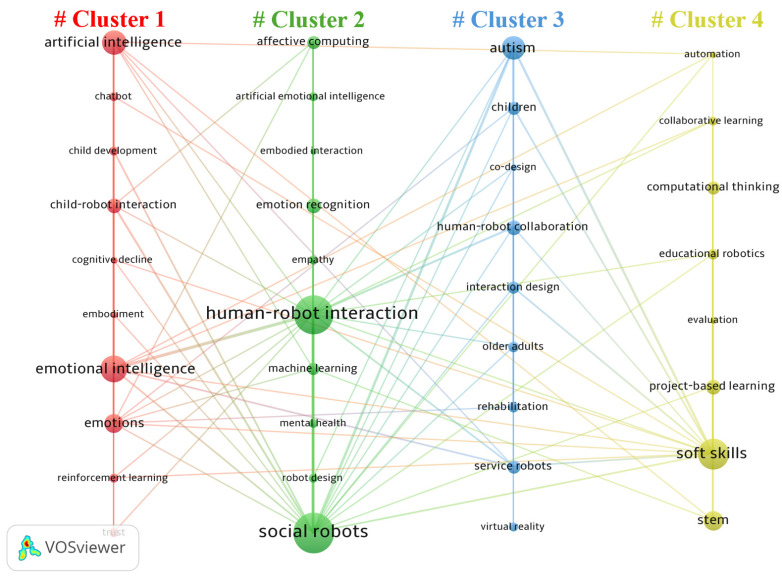
Keyword co-occurrence network of robot-assisted SEL research.

For this analysis, author-identified keywords were used, comprising 771 terms. To better visualize the commonly co-occurring keywords, the analysis was set to include keywords with a minimum of two co-occurrence cases. This reduced noise from isolated single-occurrence terms and improved network clarity by emphasizing more recurrent and structurally significant themes within the field. Using VOSviewer, the 38 most frequently co-occurring keywords were selected for demonstration. In the resulting keyword co-occurrence map (see Figure 6), the size of each circular node corresponds to the frequency of the keyword’s occurrence, reflecting its importance as a prominent topic in the field of robot-assisted SEL. Additionally, the thickness of the lines between nodes reflects the strength of their association, with thicker lines indicating a higher frequency of co-occurrence between two keywords in an article. Overall, four clusters of co-occurring keywords were distinguished by different colors.

**Cluster 1. AI-mediated socio-emotional development in child–robot relationships**. This cluster focuses specifically on children’s socio-emotional development through interaction with AI-powered robots. Keywords such as *child development*, *child–robot interaction*, *emotions*, *emotional intelligence*, and *trust* indicate research examining how children form emotional bonds, develop social understanding, and build relational trust with embodied intelligent agents. The presence of *artificial intelligence*, *chatbot*, and *reinforcement learning* suggests adaptive systems that personalize responses to support emotional growth. This cluster is fundamentally developmental and relational, centered on how AI shapes children’s emotional learning processes.

**Cluster 2. Emotion-aware social robot design for empathy and mental health support**. This cluster centers on the technological and design mechanisms that enable robots to recognize, interpret, and respond to human emotions. Keywords such as *affective computing*, *artificial emotional intelligence*, *emotion recognition*, and *machine learning* point to the computational backbone of socio-emotional responsiveness. The inclusion of *empathy*, *embodied interaction*, and *human–robot interaction* suggests that researchers are concerned not just with detecting emotion, but with creating emotionally meaningful interactions. Meanwhile, *mental health* and *social robots* indicate applied contexts where robots are deployed to provide emotional support or psychological intervention.

**Cluster 3. Inclusive and therapeutic human–robot collaboration across the lifespan**. This cluster represents application-oriented interventions, particularly in special education and healthcare contexts. Keywords such as *autism*, *rehabilitation*, and *older adults* indicate populations with specific socio-emotional or cognitive needs. The presence of *co-design*, *interaction design*, and *human–robot collaboration* suggests participatory and context-sensitive development. *Virtual reality* signals multimodal therapeutic environments. The appearance of *service robots* and *virtual reality* suggests integration of multimodal technologies in support environments. Overall, this cluster focuses on targeted interventions for specific populations, including but not limited to children with autism.

**Cluster 4. Educational robotics for SEL skill development in STEM learning**. This cluster reflects the integration of robotics into formal educational settings to foster social and emotional learning competencies. Keywords such as *educational robotics*, *collaborative learning*, and *project-based learning* indicate structured classroom activities where students work together through hands-on robotic tasks. The presence of *soft skills* directly connects this cluster to SEL components such as communication, teamwork, self-regulation, and problem-solving. Meanwhile, *evaluation* signals empirical efforts to assess the effectiveness of robotics-based SEL interventions. Although *STEM*, *computational thinking*, and *automation* appear in the cluster, they function primarily as instructional contexts rather than the central focus. In this body of research, robotics serves as a pedagogical medium through which social interaction, collaboration, and emotional competencies are cultivated and assessed.

#### 3.3.3. Thematic Evolution

**Figure 7:**
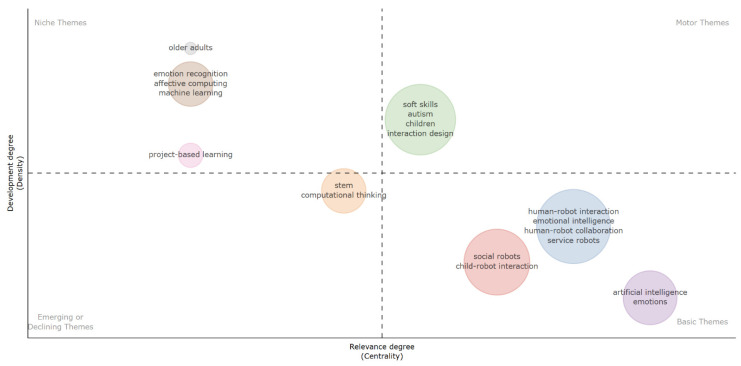
Thematic map of robot-assisted SEL research.

To gain a deeper understanding of the current state of robot-assisted SEL research and its future direction, a thematic analysis was conducted. This analysis involved examining clusters of authors-defined keywords and their interconnections to identify underlying themes. The thematic map was produced using the *bibliometrix* package in R. As shown in Figure 7, each bubble on the map represents a cluster of themes, with the size of the bubble indicating the frequency of occurrence of the corresponding keywords. These themes are characterized by two key parameters: centrality and density. Centrality measures the degree of interaction between different themes, indicating a theme’s influence within the broader research field. Density, on the other hand, measures the strength of internal connections among all the keywords within a theme, signifying its level of development. These two properties provide insights into the development and significance of specific themes or topics.

The Louvain algorithm was selected for the analysis of thematic evolution because it offers strong interpretability and robust performance for network-based thematic analysis. Compared with alternatives such as Infomap and Leiden, Louvain is computationally efficient, widely used in bibliometric studies, and produces intuitively interpretable hierarchical community structures that align well with thematic mapping purposes. Nine themes were clustered (see Figure 7) across four quadrants: motor themes, niche themes, emerging or declining themes, and basic themes.

The Motor Themes quadrant exhibits high centrality and density scores, indicating their full development and crucial role in robot-assisted SEL research. In this quadrant, the cluster comprising *soft skills*, *autism*, *children*, and *interaction design* appears both highly central and well-developed, indicating that child-focused socio-emotional skill development, particularly in special education contexts, currently drives the field. These themes are conceptually well-established and structurally prominent within the field.

The Niche Themes quadrant has a high density but low centrality, suggesting that these themes are well-developed but specialized in the field of robot-assisted SEL. In this quadrant, *emotion recognition*, *affective computing*, and *machine learning* represent technically mature yet specialized research streams that remain peripheral to the main educational discourse. The presence of *older adults* indicates population-specific applications outside the dominant child focus. Notably, *project-based learning* also appears here, suggesting that while it is used to foster soft skills such as communication and collaboration, it remains a context-specific approach rather than a central driver of robot-assisted SEL research.

The Emerging or Declining Themes Quadrant has low density and centrality values, indicating they are either emerging or declining. In this quadrant, *STEM*, *computational thinking*, and *project-based learning* exhibit lower centrality and density, suggesting that the integration of robotics-based SEL into broader curricular and pedagogical frameworks remains either nascent or not yet fully stabilized.

The Basic Themes Quadrant has high centrality and low density, indicating that they are general topics that are relevant across different research areas of robot-assisted SEL but are not fully developed. In this quadrant, large clusters such as *human–robot interaction*, *emotional intelligence*, *human–robot collaboration*, *service robots*, *social robots*, *child–robot interaction*, and *artificial intelligence* with *emotions* show high centrality but lower density. This suggests that they form the foundational conceptual backbone of robot-assisted SEL, widely connected across studies, yet still evolving in theoretical cohesion and empirical consolidation.

Taken together, the thematic focus and evolution in the field of robot-assisted SEL research show that robot-assisted SEL is emerging as an interdisciplinary field that integrates affective AI, social robotics, and educational practice, with strong roots in child-centered and special education contexts; however, while technological capabilities and therapeutic applications are relatively advanced, their systematic integration into classroom-based SEL and coherent theoretical frameworks remains an ongoing challenge.

## 4. Discussion

This study aimed to systematically chart the development, intellectual structure, and thematic evolution of robot-assisted SEL, a growing yet fragmented interdisciplinary field spanning education, psychology, and robotics. Although research on using robots to support socio-emotional development has expanded rapidly, a comprehensive synthesis of its trajectory, collaboration patterns, and thematic structure has been lacking. To address this gap, we conducted a scientometric analysis of 241 WOS publications. The following discussion interprets the findings in relation to each research question, moving from overall research trends to intellectual foundations and thematic evolution.

### 4.1. RQ1: What Is the Overall Research Trajectory of Robot-Assisted Social–Emotional Learning Research?

The bibliometric profile and publication growth trend together indicate that robot-assisted SEL is an emerging yet consolidating interdisciplinary field characterized by steady expansion and collaborative production. The sustained annual growth rate, the relatively recent average publication age, and the marked acceleration in output after the mid-2010s suggest that the field has developed in parallel with rapid advances in social robotics and AI-driven educational technologies, consistent with broader trajectories in AI in education (Zawacki-Richter et al., 2019). The high number of co-authors per document and the limited proportion of single-authored works reflect strong interdisciplinary integration across education, psychology, computer science, and engineering, a collaboration pattern commonly observed in human–robot interaction research (Belpaeme et al., 2018). Moreover, the substantial presence of proceedings papers corresponds to dissemination norms in fast-moving technological domains, where conference venues play a central role in circulating emerging findings prior to journal consolidation (Scassellati et al., 2012). At the same time, the moderate average citation rate suggests that although visibility and output are increasing rapidly, the field has not yet formed a fully stabilized or unified theoretical core, with empirical inquiry still dispersed across therapeutic, developmental, and educational applications. Taken together, these structural indicators and the growth curve depict not merely a quantitative expansion, but a field in the midst of epistemic consolidation, gradually shifting from fragmented technological experimentation toward a more coherent and education-oriented understanding of socio-emotional development through robotic mediation.

The concentration of robot-assisted SEL research in countries such as the United States, China, and England reflects broader global patterns in AI and robotics investment, where research output strongly correlates with national research capacity, funding priorities, and technological infrastructure (Leydesdorff & Wagner, 2009). High publication volumes in these regions may be driven by well-established research ecosystems that integrate engineering, cognitive science, and educational research, enabling multidisciplinary investigations into socially assistive technologies. The comparatively lower representation from other regions suggests that these structural factors continue to shape who produces knowledge in emerging technology fields (Wagner et al., 2015). This uneven distribution also raises important theoretical and practical concerns regarding the cultural generalizability of robot-assisted SEL frameworks (Jones et al., 2026), as dominant research agendas may disproportionately reflect the educational priorities, technological assumptions, and intervention models of highly resourced countries. Consequently, the field’s future development may depend not only on technological advancement but also on broader international diversification to ensure culturally responsive and contextually adaptable models of SEL.

### 4.2. RQ2: What Are the Main Intellectual Foundations Shaping the Field of Robot-Assisted SEL?

Building on the three identified themes, the broader trajectory of robot-assisted SEL research reveals a field increasingly shaped by the tension between technological innovation and the developmental complexity of socio-emotional learning. Rather than demonstrating a straightforward expansion of robotic capability into educational effectiveness, the findings suggest that robot-assisted SEL remains deeply dependent on established developmental and pedagogical theories to achieve meaningful impact. First, the prominence of emotional recognition and social understanding themes indicates that robots are primarily being positioned within frameworks consistent with social learning theory, where socially responsive agents may serve as models, reinforcers, or structured interaction partners. However, this also raises theoretical questions about whether robot-mediated interactions can fully replicate the nuanced reciprocity, cultural context, and interpersonal variability central to human-centered SEL, suggesting that robots may function more effectively as scaffolding tools than as substitutes for authentic social learning environments (Breazeal et al., 2016). Second, the strong focus on relational and affective mechanisms reflects growing scholarly interest in embodiment, trust, and empathy as prerequisites for sustained socio-emotional engagement. From a theory of mind perspective, this indicates that children’s attribution of intentionality and emotional states to robots may enhance engagement, yet such engagement does not necessarily equate to deeper socio-emotional development unless supported by guided reflection and contextualized learning experiences (Lee & Lee, 2022). This distinction is critical, as it suggests that relational sophistication alone may risk prioritizing technological simulation over substantive developmental outcomes. Third, the expansion of structured interventions and educational integration highlights a gradual shift toward more ecologically valid applications, yet the comparatively peripheral status of broader classroom implementation suggests that pedagogical embedding remains underdeveloped. This pattern reinforces scaffolding and guided interaction theories, emphasizing that robots appear most effective when embedded within human-mediated instructional systems rather than operating as autonomous educational agents (Mahoney et al., 2021). Collectively, these findings suggest that the future maturation of robot-assisted SEL will depend less on increasingly sophisticated robotic technologies alone and more on the field’s ability to integrate social learning theory, developmental psychology, and instructional design into coherent socio-technical frameworks.

Regarding the regional collaboration network, although the overall network is interconnected rather than fragmented, collaboration intensity appears uneven, with a few core countries driving most international partnerships and several others occupying more peripheral positions. This suggests that robot-assisted SEL research is globally distributed but still structurally dependent on a limited number of central actors. Such a core–periphery structure is common in emerging interdisciplinary domains, where scientifically advanced nations act as knowledge hubs linking otherwise dispersed research communities (Hwang, 2008). In AI and robotics-related fields, collaboration is often anchored in countries with strong technological infrastructures and sufficient funding, reinforcing their brokerage roles in global networks (Billones et al., 2025). At the same time, the presence of regional clusters indicates gradual diffusion and diversification of research capacity, consistent with patterns observed in the globalization of science (Leydesdorff & Wagner, 2009). Overall, the findings point to an internationally connected yet hierarchically organized collaboration landscape, highlighting both the maturity of cross-border cooperation and the need for more balanced global participation in shaping the future of robot-assisted SEL research.

### 4.3. RQ3: What Emerging Themes and Thematic Evolution Can Be Identified in Robot-Assisted SEL Research?

Regarding the result of word cloud, the dominance of *human–robot interaction*, *social robots*, and *emotional intelligence* indicates that robot-assisted SEL is theoretically anchored in social robotics, where robots are conceptualized as relational agents capable of shaping children’s affective experiences (Belpaeme et al., 2018). The visibility of *affective computing* and *emotion recognition* further suggests that advances in computational emotion modeling are central to enabling adaptive socio-emotional support (D’mello & Kory, 2015). Meanwhile, the prominence of *autism* and child-focused terms reflects the field’s strong roots in developmental and therapeutic applications (Scassellati et al., 2012). More importantly, this convergence implies that robot-assisted SEL is not developing as a purely educational innovation, but as an interdisciplinary synthesis in which technological capability is being continuously translated through developmental needs and intervention demands.

As for the keyword co-occurrence, the four clusters collectively reveal a layered structure of robot-assisted SEL research, spanning developmental mechanisms, technological design, targeted interventions, and classroom implementation. Clusters 1 and 2 represent two complementary yet distinct trajectories. Cluster 1 foregrounds children’s socio-emotional development within AI-mediated relationships, emphasizing how embodied robots function as relational partners that shape emotional understanding, trust formation, and social cognition. Prior work in child–robot interaction shows that children readily attribute social agency and moral standing to robots, forming attachment-like bonds that influence prosocial behavior and emotional engagement (Woo et al., 2021). From a social learning perspective, this suggests that robots are increasingly being positioned as supplementary social models that provide consistent cues, reinforcement, and opportunities for guided imitation. In contrast, Cluster 2 shifts the analytical lens from developmental outcomes to the technical architecture of emotional responsiveness. Research in affective computing and artificial emotional intelligence focuses on enabling robots to detect, model, and respond to human affect through multimodal sensing and machine learning (D’mello & Kory, 2015). Studies on social robots for empathy and mental health further illustrate how emotionally aware systems can support well-being and therapeutic dialogue (Robinson et al., 2013; Szondy & Fazekas, 2024). Thus, while Cluster 1 centers on how children develop socio-emotionally through interaction, Cluster 2 concentrates on how robots are engineered to make such development possible.

Clusters 3 and 4 extend this foundation into applied and pedagogical domains. Cluster 3 reflects inclusive and therapeutic applications across diverse populations, particularly in autism intervention, rehabilitation, and elder care. Research demonstrates that socially assistive robots can facilitate joint attention, emotional recognition, and social communication in children with autism, often through carefully designed, structured interactions (Scassellati et al., 2012; Zhong et al., 2025). The prominence of co-design and interaction design further suggests a shift toward participatory and context-sensitive development, ensuring that robotic systems are tailored to users’ cognitive and emotional needs. Meanwhile, Cluster 4 situates robotics within formal educational environments, where robots function as pedagogical tools to cultivate SEL competencies through collaborative and project-based learning. Educational robotics has been shown to promote communication, teamwork, and self-regulation by embedding social interaction within problem-solving tasks (Ackermann et al., 2025; Suarez et al., 2023). Importantly, in this cluster, STEM serves primarily as the instructional context, while SEL competencies constitute the intended developmental outcome. The coexistence of these two clusters suggests that robot-assisted SEL is beginning to move beyond therapeutic proof-of-concept toward broader pedagogical normalization; however, the comparatively later emergence of classroom-oriented themes also indicates that educational integration still trails behind clinical and developmental applications. Together, these clusters illustrate not just an expansion of topics, but a gradual broadening of the field’s ecological scope, from individual developmental support to inclusive intervention and, more recently, to curriculum-linked educational practice.

In terms of the results about thematic evolution, the prominence of child-centered social skill development in the Motor Themes quadrant suggests that robot-assisted SEL has increasingly aligned with evidence-based intervention frameworks, particularly in autism and special education. Recent systematic reviews indicate that social robots are most consistently applied to structured socio-emotional training programs targeting communication, joint attention, and emotion understanding among children with developmental differences (Bartl-Pokorny et al., 2021; Kouroupa et al., 2022). This concentration reflects both ethical feasibility and measurable outcome structures in special education settings, where robotic interventions can be more clearly operationalized and evaluated. At the same time, the relative peripheral position of technically sophisticated themes such as affective computing and emotion recognition implies that although emotion-aware algorithms have advanced significantly, their translation into classroom-based SEL practice remains partial (Calvo et al., 2015). Similarly, the marginal integration of STEM- and project-based pedagogies suggests that while educational robotics is expanding, its systematic coupling with SEL frameworks is still theoretically underdeveloped (Toh et al., 2016). The Basic Themes cluster further indicates that foundational constructs such as human–robot interaction and artificial intelligence continue to function as enabling infrastructures rather than fully consolidated educational paradigms, echoing broader calls for stronger theoretical integration between AI systems and developmental learning sciences (Gibson et al., 2023). Collectively, these patterns suggest a field transitioning from technology-driven experimentation toward pedagogically grounded and developmentally informed models of robot-assisted SEL yet still requiring deeper curricular embedding and theoretical synthesis.

## 5. Contributions and Implications

This study has the following contributions. First, it contributes to SEL theory by showing how robot-assisted learning environments are reshaping the relational foundations of socio-emotional development through a lens consistent with social learning theory. The findings reveal a convergence of child development, autism intervention, and embodied and affective AI, suggesting that SEL is increasingly situated within hybrid interaction systems where socially assistive robots function as social agents that model behaviors, provide feedback, and scaffold interaction. From a social learning perspective, such robots can support observational learning, imitation, and reinforcement processes by offering consistent, adaptive, and responsive social cues. In this sense, this study extends traditional human-centered SEL frameworks toward a mediated developmental perspective that incorporates embodied and AI-supported interaction as additional pathways through which socio-emotional competencies may be acquired.

Second, this study advances understanding of how robotics is being incorporated into SEL research by offering a macro-level perspective that moves beyond the intervention-specific focus of prior reviews. Existing scoping and meta-analytic studies have largely demonstrated how robots can improve socio-emotional outcomes in children with autism or special needs, but they do not show how these interventions relate to the broader evolution of the field. Through bibliometric mapping, the present study reveals that robot-assisted SEL has developed unevenly across contexts, with the strongest scholarly consolidation occurring in structured child-centered and therapeutic applications, while mainstream educational and curriculum-based implementations remain less visible. This broader comparative lens constitutes a key originality of the study, as it clarifies not only where robots have been applied, but also how the field’s overall integration into SEL practice is still contextually concentrated rather than systemically widespread.

Third, this study contributes to discussions on robot-assisted SEL design by identifying thematic patterns that suggest a gap between technological development and pedagogical integration. While areas such as affective computing and emotion recognition are well-developed within the technical literature, their comparatively peripheral position in broader SEL discourse indicates that technological advancement alone may not guarantee educational centrality. These findings highlight the importance of more closely aligning technical innovation with pedagogical frameworks and developmental objectives, thereby informing future interdisciplinary efforts in the design and application of robot-assisted SEL systems.

In addition, the findings of this study also carry the following implications. First, for practice, the findings suggest that robot-assisted SEL should be implemented as structured, curriculum-aligned interventions rather than as standalone technological activities. Educators should embed robots within clearly defined socio-emotional objectives (e.g., emotion regulation, communication, or empathy) and maintain active teacher facilitation to scaffold interaction and contextualize learning. Schools adopting robotic systems should prioritize developmental appropriateness, measurable SEL outcomes, and alignment with existing instructional frameworks to ensure sustainability beyond pilot experimentation.

Second, for research, the uneven thematic development indicates the need for stronger theoretical and methodological consolidation. Future studies should move beyond short-term feasibility trials toward longitudinal, ecologically valid classroom research that examines transfer effects and sustained socio-emotional growth. Greater attention should also be given to diverse populations, cross-cultural contexts, and comparative designs that clarify the distinct contribution of robotic mediation relative to human-led SEL interventions.

Third, for design, the results highlight the importance of aligning technological sophistication with pedagogical intent. Developers should adopt interdisciplinary co-design approaches involving educators and developmental experts to ensure that affective computing capabilities translate into meaningful socio-emotional scaffolding. Future robot-assisted SEL systems should support triadic interaction models, integrating teachers, peers, and robots, rather than relying solely on dyadic child–robot exchanges, thereby enhancing relational continuity and instructional coherence.

## 6. Limitations and Future Research Directions

First, this study relies on a single bibliographic database, which, despite its high quality and rigorous indexing standards, may underrepresent relevant non-English publications and emerging interdisciplinary work. This selection may therefore introduce coverage bias and shape the observed research landscape. Future studies should incorporate multiple databases (e.g., Scopus, IEEE Xplore) and grey literature to enhance representativeness and reduce potential bias. Second, bibliometric analyses depend on author-provided keywords and citation structures, which may not fully capture theoretical nuance, methodological diversity, or the quality of empirical evidence. As a result, thematic clustering reflects structural and conceptual patterns rather than evaluating intervention effectiveness or theoretical coherence. Future research should complement bibliometric approaches with systematic reviews or meta-analyses to critically assess research design, outcome measures, and effect sizes. Third, the quantitative science-mapping approach adopted here captures macro-level trends but cannot account for micro-level interaction processes or contextual implementation dynamics. Consequently, it does not reveal how robot-assisted SEL operates in authentic educational settings. Future research should employ qualitative or mixed-method designs to examine teacher mediation, student engagement, and contextual variability in real-world classroom environments.

## Figures and Tables

**Table 1:** General information about the dataset.

Description	Results
MAIN INFORMATION ABOUT DATA	
Timespan	2002:2026
Sources (Journals, Books, etc.)	194
Documents	241
Annual Growth Rate %	7.75
Document Average Age	5.01
Average citations per doc	14.17
References	10,175
DOCUMENT CONTENTS	
Keywords Plus (ID)	378
Author’s Keywords (DE)	831
AUTHORS	
Authors	933
Authors of single-authored docs	23
AUTHORS COLLABORATION	
Single-authored docs	24
Co-Authors per Doc	4.11
International co-authorships %	20.75
DOCUMENT TYPES	
article	108
article; early access	2
article; proceedings paper	2
proceedings paper	109
review	16
review; early access	4

**Table 2:** Top 10 most cited papers.

Rank	Author	Title	Journal	Year	LC	GC	LC/GC
1	Cabibihan et al.	Why robots? A survey on the roles and benefits of social robots in the therapy of children with autism	International Journal of Social Robotics	2013	10	383	2.61
2	Marino et al.	Outcomes of a robot-assisted social–emotional understanding intervention for young children with autism spectrum disorders	Journal of Autism and Developmental Disorders	2020	6	74	8.11
3	Giannopulu & Pradel	From child-robot interaction to child-robot-therapist interaction: A case study in autism	Applied Bionics and Biomechanics	2012	3	15	20.00
4	Pop et al.	Can the social robot Probo help children with autism to identify situation-based emotions? A series of single-case experiments	International Journal of Humanoid Robotics	2013	3	45	6.67
5	Leite et al.	Emotional storytelling in the classroom: Individual versus group interaction between children and robots	ACM IEEE International Conference on Human–Robot Interaction	2015	3	87	3.45
6	Fan et al.	Do we need emotionally intelligent artificial agents? First results of human perceptions of emotional intelligence in humans compared to robots	Intelligent Virtual Agents	2017	3	25	12.00
7	Mengoni et al.	Feasibility study of a randomised controlled trial to investigate the effectiveness of using a humanoid robot to improve the social skills of children with autism spectrum disorder (Kaspar RCT): A study protocol	BMJ Open	2017	3	40	7.50
8	So et al.	A robot-based play-drama intervention may improve the joint attention and functional play behaviors of Chinese-speaking preschoolers with autism spectrum disorder: A pilot study	Journal of Autism and Developmental Disorders	2020	3	47	6.38
9	Kewalramani et al.	Using robotic toys in early childhood education to support children’s social and emotional competencies	Australasian Journal of Early Childhood	2021	3	32	9.38
10	Kim & Tscholl	Young children’s embodied interactions with a social robot	Educational Technology Research and Development	2021	3	28	10.71

Note. LC = Local citations; GC = Global citation.

## Data Availability

The data presented in this study are available on request from the corresponding author.

## Supplementary Materials

The following supporting information can be downloaded at: https://www.mdpi.com/article/10.3390/bs16050746/s1, Table S1. Coding scheme for systematic review.
