# Supplementary material for: Charting the Development of Robot-Assisted Social–Emotional Learning: Mapping Its Intellectual Foundations, Thematic Foci, and Evolution
Source: Behav Sci (Basel). 2026 May 11;16(5):746. doi: 10.3390/bs16050746 (PMC13203568; doi:10.3390/bs16050746)
Supplement: Supplementary file 1 [file behavsci-16-00746-s001.zip › behavsci-4231633-supplementary.pdf]

Table S1. Coding scheme for systematic review

Code	Explanation of the Code
**Emotion Recognition Support**	Robot systems that help children identify, label, or respond to emotions through multimodal cues (facial expressions, tone, gestures).
**Empathy and Prosocial Skill Development**	Robots designed to foster empathy, perspective-taking, or cooperative social behaviors.
**Engagement and Social Presence**	Research examining how robot embodiment, anthropomorphism, or interaction style enhances engagement and relational bonding.
**Instructional Integration**	Studies focusing on how robots are embedded into curriculum and classroom practices for SEL.
**Adaptive Emotional Personalization**	Use of AI-driven adaptation to tailor emotional feedback or interaction patterns based on student responses.
**Teacher Mediation and Facilitation**	Research highlighting the teacher's role in orchestrating or moderating robot-assisted SEL activities.
**Ethical and Developmental Safeguards**	Consideration of ethical risks, attachment concerns, privacy, or developmental appropriateness.
**Trust and Relational Dynamics**	Studies exploring how trust formation influences SEL outcomes in human–robot interaction.
**Multimodal Interaction Design**	Use of speech, gesture, gaze, and physical embodiment to support emotional communication.
**Longitudinal Social-Emotional Impact**	Research assessing sustained SEL development over extended interactions with robots.
